# beta-Catenin Regulates Intercellular Signalling Networks and Cell-Type Specific Transcription in the Developing Mouse Midbrain-Rhombomere 1 Region

**DOI:** 10.1371/journal.pone.0010881

**Published:** 2010-06-03

**Authors:** Dmitri Chilov, Natalia Sinjushina, Jonna Saarimäki-Vire, Makoto M. Taketo, Juha Partanen

**Affiliations:** 1 Institute of Biotechnology, University of Helsinki, Helsinki, Finland; 2 Department of Biosciences, University of Helsinki, Helsinki, Finland; 3 Department of Pharmacology, Graduate School of Medicine, Kyoto University, Kyoto, Japan; Medical College of Georgia, United States of America

## Abstract

β-catenin is a multifunctional protein involved in both signalling by secreted factors of Wnt family and regulation of the cellular architecture. We show that β-catenin stabilization in mouse midbrain-rhombomere1 region leads to robust up-regulation of several Wnt signalling target genes, including Fgf8. Suggestive of direct transcriptional regulation of the Fgf8 gene, β-catenin stabilization resulted in Fgf8 up-regulation also in other tissues, specifically in the ventral limb ectoderm. Interestingly, stabilization of β-catenin rapidly caused down-regulation of the expression of Wnt1 itself, suggesting a negative feedback loop. The changes in signal molecule expression were concomitant with deregulation of anterior-posterior and dorso-ventral patterning. The transcriptional regulatory functions of β-catenin were confirmed by β-catenin loss-of-function experiments. Temporally controlled inactivation of β-catenin revealed a cell-autonomous role for β-catenin in the maintenance of cell-type specific gene expression in the progenitors of midbrain dopaminergic neurons. These results highlight the role of β-catenin in establishment of neuroectodermal signalling centers, promoting region-specific gene expression and regulation of cell fate determination.

## Introduction

Wnts are a family of secreted lipoproteins, which play a crucial role during embryogenesis via the regulation of patterning, cell fate decision and cell polarity. Wnts mediate their intracellular effects by inducing stabilization and nuclear translocation of β-catenin. In the absence of Wnt ligands, cytoplasmic β-catenin is phosphorylated by glycogen synthase kinase 3β (GSK3β). Phosphorylated β-catenin is targeted for ubiquitination and proteasomal degradation. Binding of Wnt molecules to their cell surface receptors releases β-catenin from the destruction complex followed by accumulation and nuclear translocation of β-catenin. Nuclear β-catenin complexes with the TCF/LEF family of transcription factors to regulate Wnt target gene expression (reviewed in [1]).

During development of CNS Wnt signalling acts as important posteriorizing factor and correct anterior-posterior (AP) patterning requires anterior inhibition of Wnt pathway [2], [3]. At the cellular level, canonical Wnt signalling endorses mitogenic pathway. While β-catenin maintains proliferation, [4], inactivation of β-catenin accelerates expression of neurogenic genes [5] and causes premature neuronal differentiation [6]. On the other hand, overexpression of stabilized β-catenin in cortical precursors leads to increased cell cycle re-entry and subsequent overproduction of neurons [7].

In the midbrain, early Wnt activity is responsible for establishment of a local organizing centre, the isthmic organizer [8]. Inactivation of Wnt signalling via Wnt1 or β-catenin gene ablation results in the deletion of posterior midbrain and part of cerebellum [9], [10], [11]. Wnt family members also play multiple roles in generation of midbrain dopaminergic neurons *in vivo* and *in vitro* [12], [13], [14].

In this work we studied the role of β-catenin in neuronal development in the midbrain. For this, we applied spatially and temporally controlled stabilization and inactivation of β-catenin in mouse embryos. Our findings underscore β-catenin as important transcriptional co-factor regulating midbrain gene expression and patterning.

## Materials and Methods

### Generation and genotyping of mice and embryos

Generation and genotyping of an *Engrailed 1 (En1)* allele carrying Cre-recombinase knock-in [15], Rosa26 locus carrying tamoxifen inducible *R26cre-ert* [16], conditional β*-catenin* loss-of-function allele [11], conditional β*-catenin* loss-of-exon3 allele [17] and transgenic mice expressing LacZ gene under control of β-catenin/TCF responsive elements [18] were described elsewhere. For staging, the day of vaginal plug was counted as embryonic day 0.5 (E0.5). To induce Cre-recombinase in *R26cre-ert* mice, pregnant females were given intraperitoneal injection of tamoxifen (Sigma) (8 mg/40 g body weight). All animal work has been conducted according to relevant national and international guidelines. Approval has been obtained from the Finnish Committee of Experimental Animal Research.

### *In situ* mRNA hybridization

Whole-mount mRNA *in situ* analysis was performed by a modified protocol [19] using a digoxigenin-labeled antisense probes. Radioactive mRNA *in situ* hybridization on paraffin sections was performed as described previously [20] using ^35^S-labeled antisense probes. Probes used were: *Drapc1* [21], *Fgf8* [22], *Otx2* [23], *Aldh1a1* [24], *Pax3* (IMAGE RZPDp981A09196D), *Ngn2* (IMAGE 2922473), *Lmx1a* (IMAGE 317647), *Phox2a* (IMAGE 480100), *Mash1* (IMAGE 6415061), *Wnt1* (gift from Klaus Schughart), *Shh* (gift from Andrew McMahon), *Gli1* (gift from David Rice), *Axin2, Tcf1, Dkk1* (gift from Irma Thesleff).

### Immunofluorescence

Immunfluorescent staining on paraffin sections was performed as described previously [25]. Primary antibodies used were mouse monoclonal against β-catenin, (BD South San Francisco, CA) and rabbit polyclonal against Lmx1a (a gift from Michael German), tyrosine hydroxylase (Chemicon) and Aldh1a1 (Abcam, Cambridge, UK).

### Microscopy

Whole-mount staining was visualized with a Leica MZFLIII microscope and photographed using an Olympus DP50-CU camera. Staining on paraffin sections were visualized with an Olympus AX70 microscope and photographed using an Olympus DP70 camera. Images were processed and assembled using Adobe Photoshop software. Confocal images were acquired using the Leica TCS SP5 confocal system and LAS-AF software. Confocal stacks and images were processed and deconvoluted using Imaris 6.1 (Bitplane) and AutoQuantX (AutoQuant) software.

## Results

To study the role of β-catenin in midbrain neurogenesis we used conditional stabilization and inactivation of β-catenin. In the *En1* ^cre/+^; *β-catenin* ^flox(ex3)/+^ embryos excision of exon 3, containing the GSK3β phosphorylation sites, prevents targeting of β-catenin for proteosomal degradation and is expected to lead to β-catenin protein accumulation in the midbrain and rhombomere1 (r1) [17]. Conditional inactivation of β*-catenin* in *R26* ^cre-ert/+^; *β-catenin* ^flox/flox^ embryos was induced by intraperitoneal injection of tamoxifen into pregnant dams [11]. Tamoxifen activates the Cre-Ert2 fusion protein, which in turn recombines and inactivates the *β-catenin* ^flox^ allele. In the following, we call the *En1* ^cre/+^; *β-catenin* ^flox(ex3)/+^ and *R26* ^cre-ert/+^; *β-catenin* ^flox/flox^ embryos as β-catenin^stab^ (stabilized) and β-catenin^lof^ (loss of function), respectively.

### β-catenin stabilization leads to profound up-regulation of Wnt target gene expression

**Figure 1:**
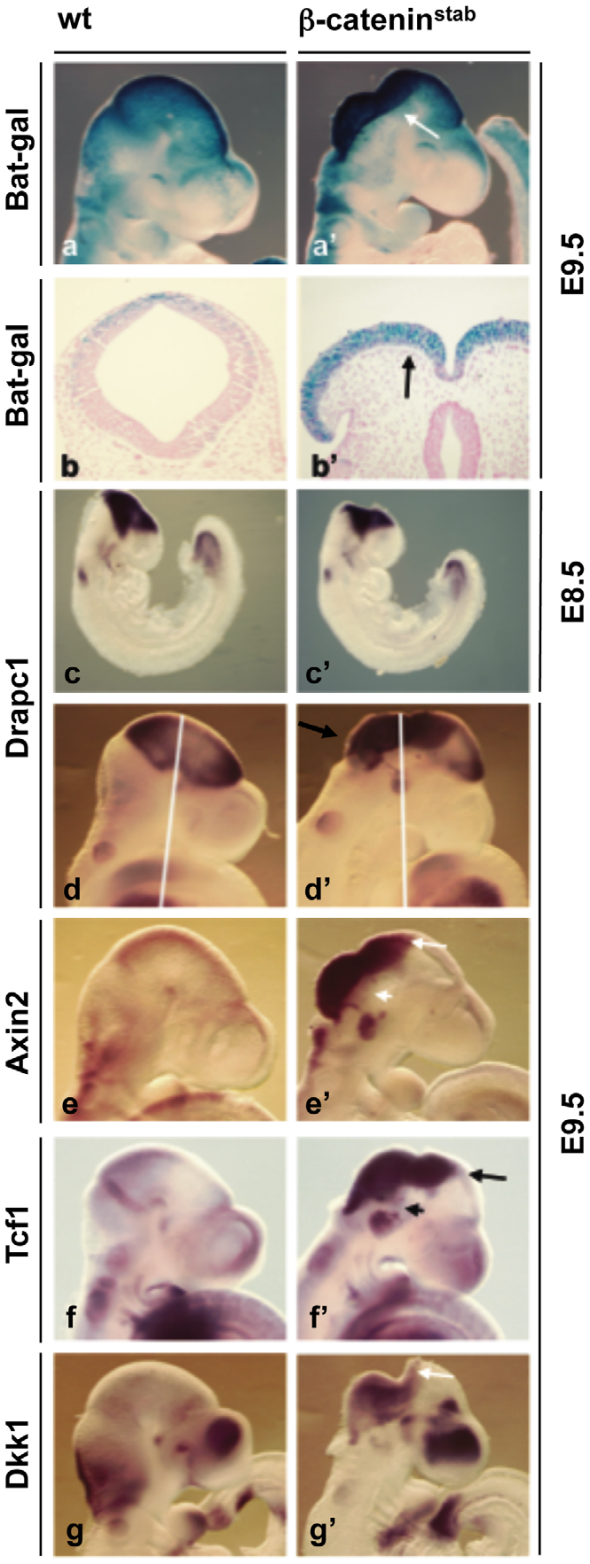
Effect of β-catenin stabilization on Wnt target gene expression. β -galactosidase staining on whole mount (a,a') and coronal section (b,b') and whole mount *in situ* hybridization (c–g**'**) of E8.5 (c–c') and E9.5 (d–g**'**) wt (a–g) and β-catenin^lof^ (a'–g') embryos. Probes were against *Drapc1*, *Axin2*, *Tcf1*, *Dkk1*. Arrows indicate changes in gene expression. Arrowheads point to down-regulation of *Axin2* and *Tcf1* in ventral midbrain.

**Figure 2:**
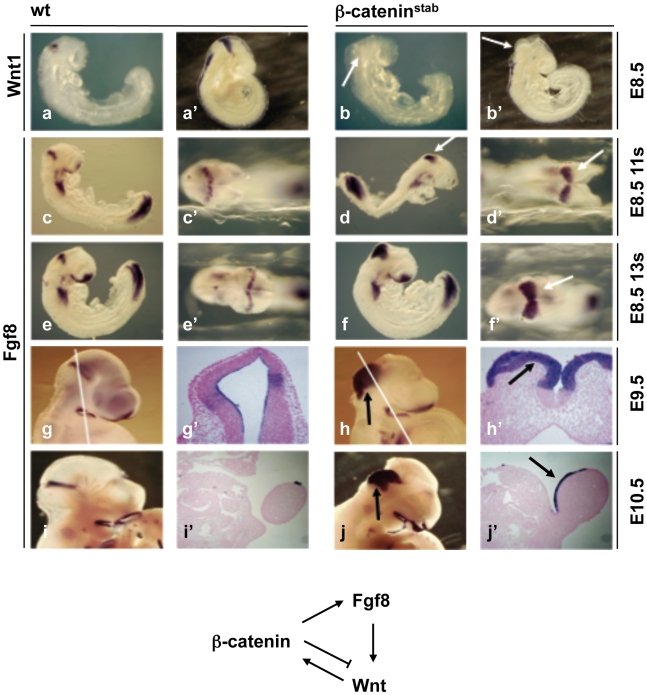
Effect of β-catenin stabilization on isthmic gene expression. Whole-mount (a–g, h, i,j) and section (g',h',i',j') *in situ* hybridyzation of E8.5 (a–f'), E9.5 (g–h') and E10.5 (i–j') wt and β-catenin^stab^ embryos for *Wnt* (a–b') and *Fgf8* (c–j') expression. Straight line in g, and h defines position of coronal section shown in g'and h' respectively. Arrows indicate changes in gene expression. Whole mount *in situ* images show lateral c,d, e,f and dorsal c',d',e',f' views of the same embryos. The scheme represents a model of signal interactions in rhombomere1/midbrain region. β-catenin stimulates expression of *Fgf8* but simultaneously inhibits *Wnt1* expression itself.

To verify β-catenin stabilization in the β-catenin^stab^ embryos, we crossed transgenic mice expressing LacZ gene under control of β-catenin/TCF responsive elements (BAT-gal) with β-catenin^flox(ex3)^ mice [18]. In BAT-gal; β-catenin^stab^ embryos, β-galactosidase staining was drastically increased in rostral rhombomere1 and caudal midbrain – correlating with the domain of *Engrailed 1* expression (**Fig. 1 a,b**). Intriguingly, in β-catenin^stab^ embryos neural tube fails to close in the midbrain-rhombomere1 region (Fig. 1b**′** and **Fig. 2h′**). To analyse effect of β-catenin stabilization on target gene expression, we carried out whole mount *in situ* hybridization using probes against direct targets of β-catenin including *Tcf1*, *Dkk1*, *Axin2* and *Drapc1*. At E8.5 *Drapc1* expression was confined to the midbrain and diencepalon (Fig. 1c,c**′**). A day later at E9.5, *Drapc1* was drastically up-regulated in the dorsal midbrain and also expressed ectopically in the dorsal hindbrain of β-catenin^stab^ embryos (Fig. 1d,d′). *Axin2*, which was absent from rhombomere1 in wt (wt) embryos, was strongly induced in dorsal midbrain and rhombomere1 in β-catenin^stab^ embryos (Fig. 1e,e′). Profound increase in *Tcf1* and *Dkk1* mRNA levels was also observed in dorsal midbrain-rhombomere 1 region (Fig. 1f–g′). Taken together, these results indicate that transcriptional targets of β-catenin are intensely up-regulated upon β-catenin stabilization in rhombomere1 and midbrain.

### Effect of β-catenin stabilization on Fgf8 and Wnt1 expression at the isthmic organizer

Using tissue-electroporation of chick embryos, induction of *Fgf8* by *Wnt1* was demonstrated in the anterior hindbrain [26], [27]. However, in mouse embryos ectopically expressing Wnt1, only subtle caudal expansion of the *Fgf8* expression domain in the hindbrain was reported [28]. Suggesting regulation of *Fgf8* expression by the canonical Wnt pathway, we observed strong up-regulation and caudal spreading of *Fgf8* expression in the hindbrain of β-catenin^stab^ embryos already at E8.5 (Fig. 2d,d′,f, f′). At E9.5-E10.5, the *Fgf8* expression encompassed the whole rhombomere1 and was highly up-regulated (Fig. 2h,h′, j). Interestingly, *Fgf8* expression in the limb bud of was expanded to cover the ventral limb ectoderm where *En1* ^Cre^ also drives recombination of the *β-catenin* ^flox(ex3)^ allele and stabilization of the β-catenin protein in the β-catenin^stab^ embryos (Fig. 2j′). This cell context independence suggests a rather direct regulation of *Fgf8* gene expression by β-catenin.

In turn, Fgf8 is important to maintain expression of Wnt1 in the early mouse midbrain [29] and Fgf8 beads induce expression of *Wnt1* in chick embryos [30]. Therefore, we studied how over-expression of Fgf8 in β-catenin^stab^ embryos affects Wnt1 expression. Surprisingly, Wnt1 mRNA was undetectable in the midbrain of β-catenin^stab^ embryos already at E8.5 (Fig. 2b,b′). Thus, in addition to the positive feedback-loop through Fgf8, Wnt1 also appears to directly negatively regulate its own expression through β-catenin.

### β-catenin stabilization results in anterio-posterior patterning defects

**Figure 3:**
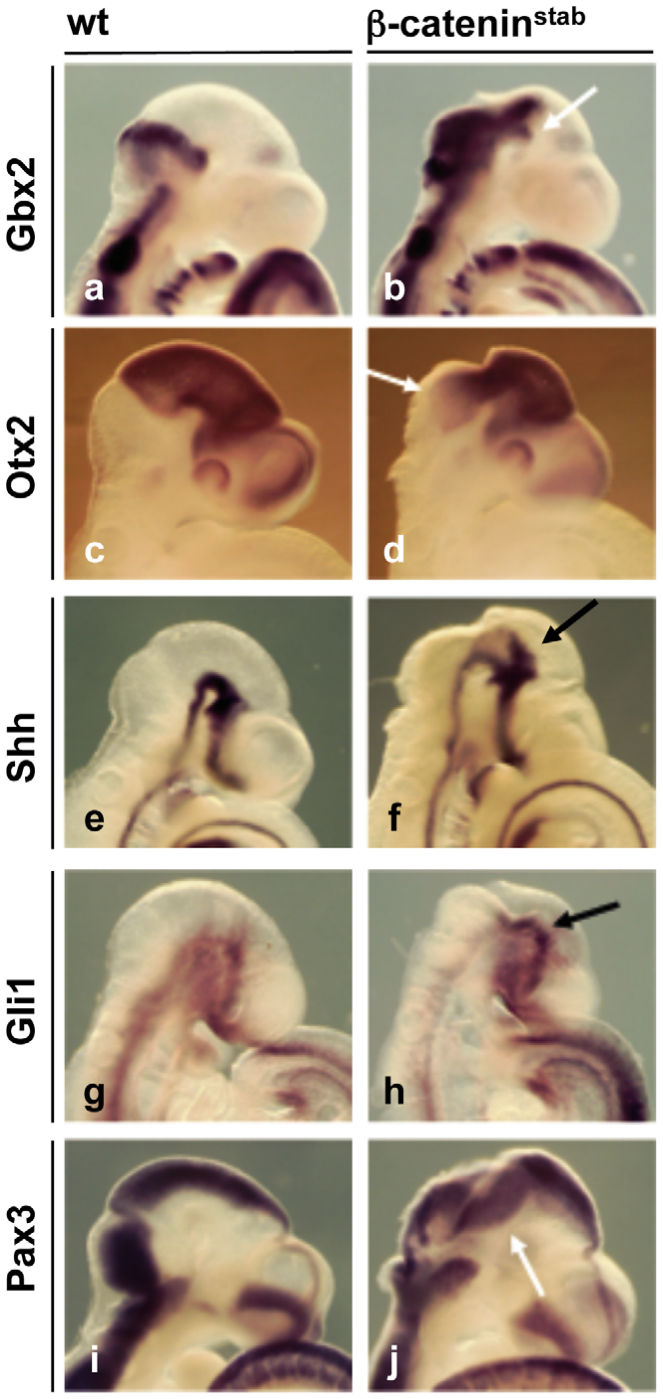
De-regulation of anterior-posterior and dorso-ventral patterning upon β-catenin stabilization. Whole mount *in situ* hybridyzation of E9.5 (22–26 somites) wt (a,c,e,g,i) and β-catenin^stab^ (b,d,f,h,j) embryos using probes against *Gbx2* (a,b), *Otx2* (c,d), *Shh* (e,f), *Gli1* (g,h), *Pax3* (i,j). Arrows indicate changes in gene expression. In g and h, the embryos have 21–22 somites.

In the β-catenin^stab^ embryos, strong β-catenin signalling and up-regulation of Fgf8 can be expected to cause defects in antero-posterior patterning. Indeed, *Gbx2* expression is enhanced and extended into anterior midbrain in the β-catenin^stab^ embryos (**Fig. 3a,b**). Despite expansion of the *Gbx2* expression, *Otx2* is not down-regulated in the midbrain but it instead is ectopically expressed in the rhombomere1 (Fig. 3c,d). Taken together, these results indicate that β-catenin regulates anterior-posterior patterning directly by promoting midbrain fate and indirectly by activating Fgf8. Activation of both β-catenin target genes and Fgf8 pathway allows co-expression of Gbx2 and Otx2 and disrupts midbrain-hindbrain boundary formation in β-catenin^stab^ embryos.

### β-catenin stabilization affects dorso-ventral patterning and neurogenesis in the midbrain

Wnt family members are expressed in the ventral midbrain as well as dorsal roof plate. Thus, transcriptional regulation by β-catenin may affect dorso-ventral patterning either directly or through other signalling molecules. In β-catenin^stab^ embryos, the ventralizing morphogen *Shh*, normally expressed in the midbrain floor plate and basal plate, expanded from the ventral midbrain towards the dorsal midbrain (Fig. 3e,f). This shift was most prominent in the anterior midbrain negative for ectopic *Gbx2* activation (see above). Consistent with the spreading of the Shh, expression of *Gli1*, a target and a mediator of Shh signalling, was enhanced in the dorsal midbrain of β-catenin^stab^ embryos (Fig. 3g,h). Despite spreading of the Shh expression, the dorsal marker *Pax3*, shown to be repressed by Shh [31] was only slightly down-regulated (Fig. 3i,j). Although in β-catenin^stab^ embryos β-catenin is activated both in the midbrain and rhombomere 1, the dorsal spreading of *Shh* and *Gli1* expression was observed only in the midbrain-like tissue.

**Figure 4:**
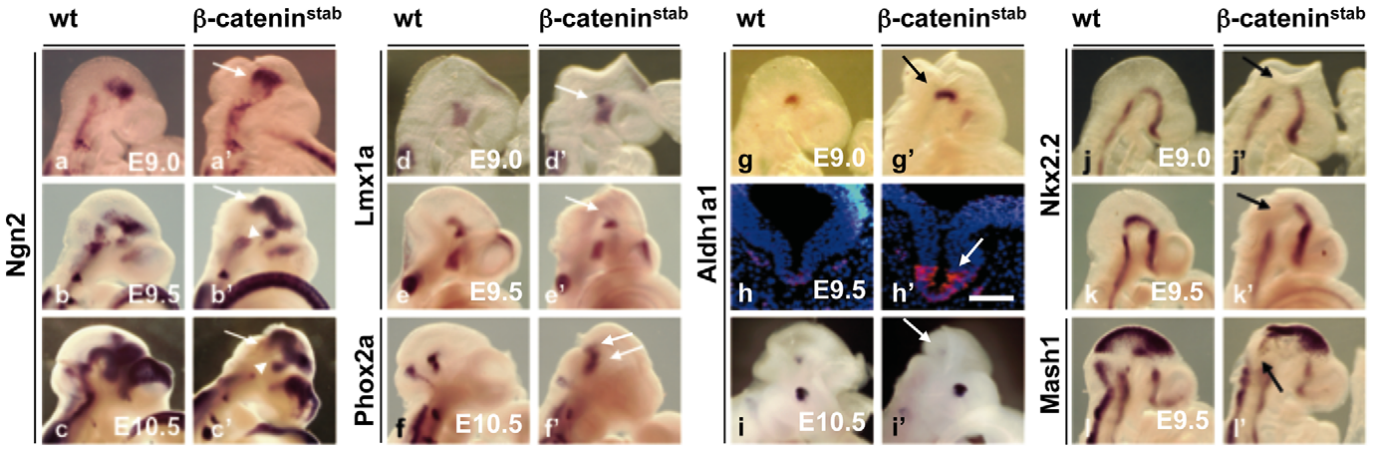
Effect of β-catenin stabilization on neurogenesis. Whole mount *in situ* hybirdyzation of E9.0 (a,d,g,j), E9.5 (b,e,h,k,l), E10.5 (c,f,i) wt and β-catenin^stab^ embryos using probes against *Ngn2* (a,b,c), *Lmx1a* (d,e), *Phox2a* (f), *Aldh1a1* (g,i), *Nkx2.2* (j,k) and *Mash1* (l). Immunofluorescent analysis of Aldh1a1 expression on coronal section of wt and β-catenin^stab^ embryos (h, Aldh1a1 with red colour, nuclear dapi staining blue colour). Arrows in a,b,c mark ectopic dorsal expression of *Ngn2*, arrowhead shows down-regulation of *Ngn2* ventrally. Other arrows indicate altered gene expression. Scale bar: 100 µm.

Similar to dorsal expansion of *Shh* and *Gli1*, we observed early up-regulation and dorsal expansion of several other ventrally expressed genes. However, the ventral up-regulation was often transient and was followed by apparently complete inactivation of expression in the ventral tissue. These genes included *Ngn2*, which was up-regulatedin the midbrain of β-catenin^stab^ embryos at E9.0 (**Fig. 4a,a′**). In contrast, high *Ngn2* levels in the dorsal midbrain were paralleled by a dramatic decline of *Ngn2* expression in ventral rhombomere1 and midbrain of β-catenin^stab^ embryos at E9.5-10.5 (Fig. 4b–c′). Also expression of another proneural gene *Mash1* was down-regulated in the ventral midbrain (**Fig. 4l**, l′).

Similar changes were seen in markers of specific ventral neuronal populations. In the progenitors of the dopaminergic neurons developing in the most ventral midbrain, we observed up-regulation of *Lmx1a* in β-catenin^stab^ embryos at E9.0 (Fig. 4d,d′). However, this increase was temporary and reversed to *Lmx1a* down-regulation 12 hours later (Fig. 4e,e′). Similar to Lmx1a, expression of *Aldh1a1*, a specific early marker for dopaminergic neuron progenitors, was up-regulated in ventral domain of β-catenin^stab^ embryos at E9.0 (Fig. 4g,g′,h,h′) but decreased by E10.5 (Fig. 4i,i′). In contrast to the dopaminergic neuron progenitor domain, *Nkx2.2* expressing progenitors were markedly reduced in the ventral midbrain of β-catenin^stab^ embryos already at E9.0 (Fig. 4j,j′,k,k′). Homeobox transcription factor *Phox2a* normally expressed in ventral motoneuron precursors of the oculomotor nucleus (III) in the midbrain and trochlear (IV) nucleus in the rhombomere 1. *Phox2a* expression was expanded to dorsal midbrain of β-catenin^stab^ embryos (Fig. 4f,f′). The continued expression of Phox2a at E9.5-E10.5 may be due to its expression in the post-mitotic precursors as opposed to the proliferative progenitors. Interestingly, the dorsal spreading of *Phox2a* expression and putative motor neuron population was seen in the midbrain but not in the rhombomere 1. This parallels the changes observed in the *Shh* expression.

Thus, the effect of β-catenin stabilization on dorso-ventral gene expression largely depends on the developmental stage. Initially, the expression of ventral markers is increased and expanded. Later, several of the ventrally expressed genes are down-regulated in their normal expression domain but can be ectopically expressed in the dorsal regions.

### β-catenin inactivation leads to the loss of transcriptional targets of Wnt signalling

**Figure 5:**
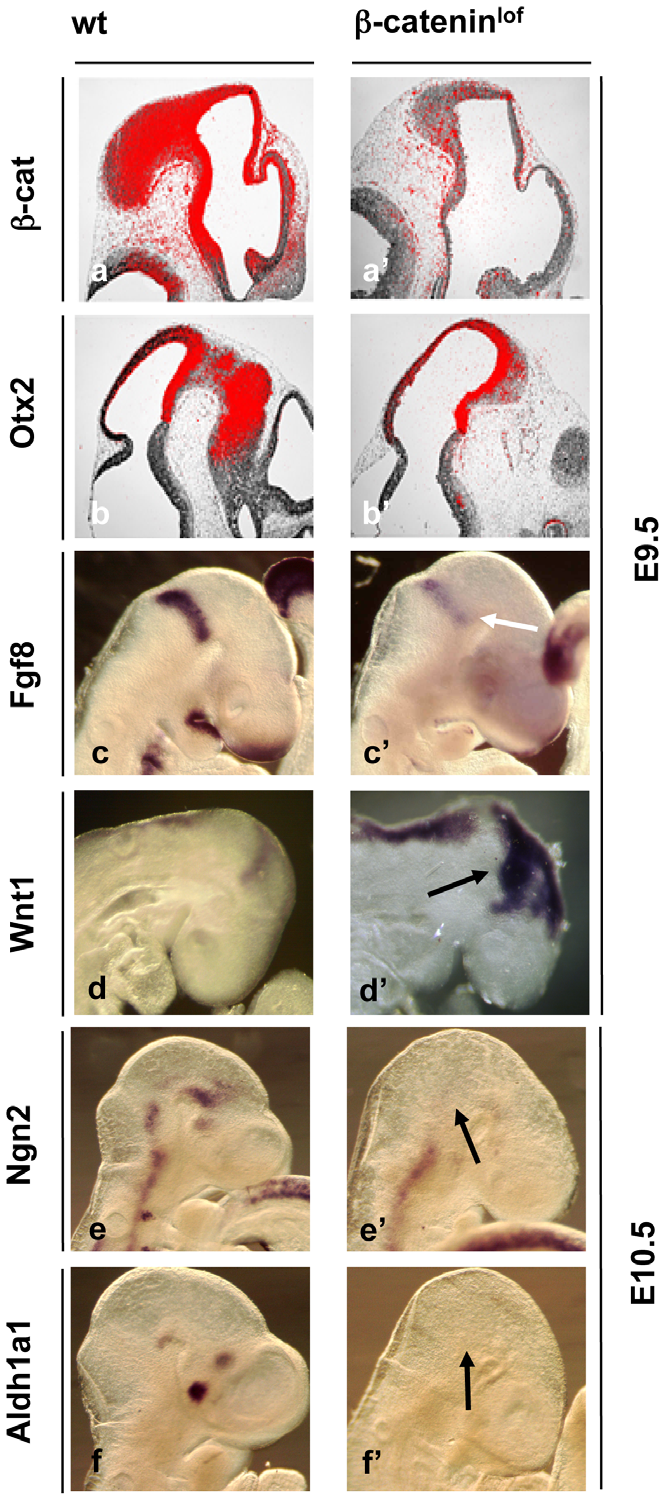
β-catenin inactivation leads to the loss of transcriptional targets of Wnt signalling. Whole mount and section *in situ* hybridization of E9.5 (a–d**'**), E10.5 (e–f**'**) wt and β-catenin^lof^ embryos using probes against β*-catenin* (a), *Otx2* (b), *Fgf8* (c), Wnt1 (d), *Ngn2* (e), *Aldh1a1* (f). Arrows indicate changes in gene expression. Tamoxifen was injected intraperitoneally into pregnant dams at E8.5, E9.5 followed by embryos isolation 24 hour later.

To validate results of β-catenin stabilization in β-catenin^stab^ embryos, we used conditional inactivation of β-catenin. The Cre recombinase was activated in *R26* ^cre-ert/+^; *β-catenin* ^flox/flox^ embryos at E8.5 or E9.5 and the effects of gene expression were analysed 1 day later at E9.5 or E10.5. The *in situ* hybridization, using probe against wild type β-catenin demonstrated significantly reduced β-catenin level throughout the neuroectoderm in β-catenin^lof^ embryos. However, some residual β-catenin mRNA persisted **(** **Fig. 5a,a′** **)**. As expected, Fgf8 mRNA was down-regulated (Fig. 5c,c′), Wnt1 mRNA was drastically up-regulated and ventrally expanded (Fig. 5d,d′) but *Otx2* expression was not significantly changed (Fig. 5b,b′) in the β-catenin^lof^ embryos. Ventral expression of *Ngn2* was abolished at E10.5 (Fig. 5e,e′). Taken together, these data and findings obtained using β-catenin stabilization corroborate the role of β-catenin as a regulator of cell identities in the midbrain. Residual expression of some of the target genes may be due to incomplete abrogation of β-catenin expression (see below).

### β-catenin inactivation distorts cell fate determination

**Figure 6:**
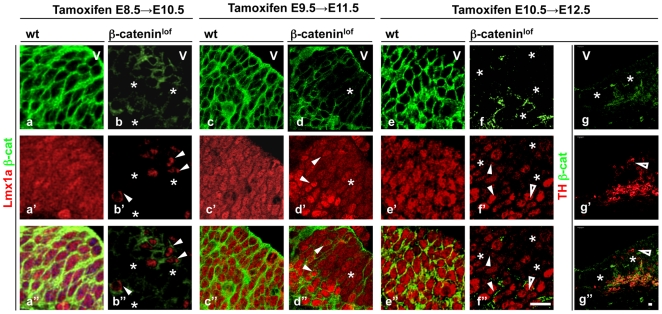
β-catenin autonomously regulates transcription in ventral neuronal progenitors. Coronal sections through midbrain of wt (a,c,e) and β-catenin^lof^ (b,d,f,g) embryos isolated at E10.5 (a,b), E11.5 (c,d) and E12.5 (e,f,g). Sections were co-stained with β-catenin (green), Lmx1a and TH (red) antibody. Asterisks mark β-catenin negative areas. Filled arrowheads indicate β-catenin+, Lmx1a+ cells in β-catenin^lof^ embryos. Cleared arrowheads point to β-catenin negative Lmx1a or TH expressing cells. V- ventricle. Tamoxifen was injected intraperitoneally into pregnant dams 48 hours prior to embryo isolation. 10 µm

We have shown previously that, consistent with incomplete inactivation of β-catenin mRNA in β-catenin^lof^ mutants, the β-catenin negative cells were scattered within β-catenin positive tissue in a mosaic pattern [32]. We exploited the mosaic β-catenin^lof^ embryos to study whether β-catenin cell-autonomously regulates transcription in the ventral dopaminergic neuron progenitors. Lmx1a protein level drastically decreased in β-catenin^lof^ embryos treated with tamoxifen at E8.5 and analysed at E10.5 (Fig. 6b′,b″). Importantly, Lmx1a protein was reduced predominantly in β-catenin protein-null cells while β-catenin positive cells of β-catenin^lof^ embryos mostly maintain normal level of Lmx1a. Lmx1a protein also decreased in large proportion of β-catenin knockout cells of β-catenin^lof^ embryos treated with tamoxifen at E9.5 (analysis at E11.5) and at E10.5 (analysis at E12.5) (Fig. 6d′,d″,f′,f″). However, a few β-catenin protein-null cells still maintained Lmx1a expression (Fig. 6f′,f″) in E12.5 β-catenin^lof^ embryos. This indicates that cell autonomous regulation of Lmx1a by β-catenin is stage dependent and as the neuronal progenitors progress towards differentiation, β-catenin – mediated control of Lmx1a is lost. In conclusion, our results suggest that β-catenin directly regulates expression of a cell-fate determining transcription factor in the proliferative dopaminergic neuron progenitors. Consistent with expression of Lmx1a in E12.5 β-catenin^lof^ embryos the dopominergic marker tyrosine hydroxylase (TH) is detected in few β-catenin protein-null cells (Fig. 6g). However, majority of β-catenin protein-null cells lack TH. Stabilization of β-catenin in the midbrain of E12.5 β-catenin^stab^ embryos completely abolishes TH expression (data not shown). This is likely due to general mispatterning of the ventral midbrain.

## Discussion

We have analyzed the role of β-catenin in development of the midbrain-rhombomere 1 region using spatially and temporally controlled stabilization and inactivation of the β-catenin locus in mice. We have shown that β-catenin regulates anterior-posterior and dorso-ventral patterning in the midbrain-rhombomere 1 region at least partly by activating expression of Fgf8 and Shh, two other important patterning signals. In addition, our results suggest direct cell-autonomous effects of β-catenin on the expression in the progenitor cells of dopaminergic neurons in the ventral midbrain.

### Cross-talk between Wnt and Fgf8 signalling

Accumulation of the degradation resistant β-catenin in the midbrain and rhombomere1 leads to drastic up-regulation of the expression of Wnt target genes, including *Drapc1, Axin2* and *Tcf1*. High level of Wnt/β-catenin signalling in β-catenin^stab^ embryos also coincides with increase and caudal expansion of *Fgf8* expression. In the wild-type embryos, Fgf8 is induced broadly in the rhombomere 1 but gets restricted to the most anterior rhombomere 1 next to the Wnt expressing posterior midbrain by E9.0. In the β-catenin^stab^ embryos, Fgf8 is expressed in the entire rhombomere 1 still at E10.5, but is not induced in the midbrain despite robust activation of the β-catenin targets. This strongly suggests that the canonical Wnt signalling pathway, involving transcriptional regulation by β-catenin, maintains *Fgf8* expression in the anterior rhombomere 1. Regulation of *Fgf8* expression by β-catenin does not appear to be restricted to the rhombomere 1. β-catenin stabilization is sufficient to turn on *Fgf8* expression in the ectoderm of ventral limb bud. Furthermore, analyses of the β-catenin^lof^ mutants indicated that in addition to the rhombomere 1, also many other domains of *Fgf8* expression are dependent on β-catenin activity. These observations together suggest a rather direct role for β-catenin in *Fgf8* regulation.

On the other hand, inactivation of Fgf8 leads to loss of *Wnt1* in midbrain-hindbrain border (reviewed in [33]. Surprisingly, *Fgf8* up-regulation does not lead to increase in *Wnt1* expression in β-catenin^stab^ mutants. On the contrary, we observe complete down-regulation of *Wnt1*. Consistently, Wnt1 was up-regulated in β-catenin^lof^ mutants. Wnt1 may by directly negatively regulated by β-catenin/TCF complex or other intermediate players regulate its expression. The β-catenin^stab^ embryos demonstrate overlapping expression of *Otx2* and *Gbx2* and consequent loss of midbrain-hindbrain border. Correspondingly, anteriorization of *Gbx2* expression could exert inhibitory effect on mesencepahlic gene expression and cause *Wnt1* down-regulation [34].

### β-catenin as transcriptional co-activator regulates patterning of the midbrain

In addition to its role in anterior-posterior patterning, Wnt/β-catenin activation has previously been shown to promote dorsal gene expression and inhibit ventral gene expression in the telencephalon and spinal cord [35], [36]. At E9.5-E10.5, this was also observed in the midbrain of β-catenin^stab^ embryos. In contrast, at E9.0, a transient elevation of several of the ventrally expressed genes, including *Ngn2*, *Aldh1a1* and *Lmx1a* was observed in the midbrain. This may reflect the fact that, unlike the spinal cord and telencephalon, the ventral midbrain has a strong endogenous Wnt signalling activity, which is used for ventral patterning [12], [37]. The reason for the eventual down-regulation of the ventral gene expression in the β-catenin^stab^ embryos remains unclear.

De-regulation of dorso-ventral patterning in β-catenin^stab^ embryos is paralleled by changes in expression of *Shh* and *Gli1* in the midbrain. Shh/Gli pathway plays a major role in ventral patterning of neural tube. In the spinal cord, the Shh pathway is antagonized by dorsally expressed Wnts by activation of the transcriptional repressor Gli3 [35]. Our results reveal somewhat different interactions of the two signalling pathways, as both *Shh* and *Gli1* expression expanded dorsally in the midbrain of β-catenin^stab^ mutants. However, we observe drastic reduction of Wnt1 also in the midbrain roof plate of β-catenin^stab^ embryos, indicating that dorsal expansion of Shh maybe due to the lack of the roof plate specific gene expression. Interestingly, the dorsal expansion of *Shh* expression was not observed in the rhombomere 1, despite stabilization of β-catenin also in this region. This may reflect the fact that *Shh* expression is restricted to the floor plate in the rhombomere 1 but it is more broadly expressed in the basal plate in the midbrain. Thus, midbrain neuroepithelium may be more competent to activate *Shh* expression.

Activation of Shh signalling in the dorsal midbrain may also be the reason for ectopic dorsal Ngn2 expression and production of Phox2a positive precursors also in the dorsal midbrain.

### β-catenin and cell fate determination

Ventral midbrain is the location where the dopaminergic neurons (DA) of substantia nigra and ventral tegmental area are generated. Lmx1a is an early and crucial activator of differentiation of ventral midbrain progenitors into DA neurons [38]. Lmx1a has been shown to be controlled by Shh. We show that Lmx1a is down-regulated in β-catenin^lof^ embryos and that β-catenin is cell autonomously required for Lmx1a expression. The loss of Lmx1a in a subpopulation of ventral midbrain progenitors suggests that Lmx1a negative cells, which were otherwise destined to become DA neurons would adopt different fate. Specification of serotonergic neurons is also controlled by a combinatorial effect of Fgf8 and Shh and it would be tempting to speculate that serotonergic neurons are generated at the expense of DA neurons. However, we could not indentify any ectopic 5'HT staining (data not shown). Our results are consistent with a recently published work showing that β-catenin signalling is required for dopaminergic neuron development in the ventral midbrain [37], [39]. Thus, in addition to regulation of other signalling pathways, β-catenin directly regulates cell fate specification of the midbrain dopaminergic neurons.

Loss of β-catenin in neuroepithelium has been shown to cause the defects in adherent junctions [39]. We have addressed the role of β-catenin in adherence in a parallel study, where we demonstrated that β-catenin maintains cell polarity and microtubules network in midbrain neural progenitor cells [32].

Taken together our results emphasize the role of β-catenin as a transcriptional co-regulator involved in anterior-posterior and dorso-ventral patterning in developing midbrain and rostral hindbrain. In part, this is achieved by interactions with other signalling pathways. We further identify β-catenin as a cell-autonomous regulator of cell fate specification in the ventral mouse midbrain.
